# Estimation and Uncertainty Analysis of Impacts of Future Heat Waves on Mortality in the Eastern United States

**DOI:** 10.1289/ehp.1306670

**Published:** 2013-11-06

**Authors:** Jianyong Wu, Ying Zhou, Yang Gao, Joshua S. Fu, Brent A. Johnson, Cheng Huang, Young-Min Kim, Yang Liu

**Affiliations:** 1 Department of Environmental Health, Rollins School of Public Health, Emory University, Atlanta, Georgia, USA; 2 Department of Civil and Environmental Engineering, University of Tennessee, Knoxville, Tennessee, USA; 3 Atmospheric Science and Global Change Division, Pacific Northwest National Laboratory, Richland, Washington, USA; 4 Department of Biostatistics and Bioinformatics, Rollins School of Public Health, Emory University, Atlanta, Georgia, USA; 5 Department of Global Health, School of Public Health and Health Services, George Washington University, Washington, DC, USA

## Abstract

Background: Climate change is anticipated to influence heat-related mortality in the future. However, estimates of excess mortality attributable to future heat waves are subject to large uncertainties and have not been projected under the latest greenhouse gas emission scenarios.

Objectives: We estimated future heat wave mortality in the eastern United States (approximately 1,700 counties) under two Representative Concentration Pathways (RCPs) and investigated sources of uncertainty.

Methods: Using dynamically downscaled hourly temperature projections for 2057–2059, we projected heat wave days that were defined using four heat wave metrics and estimated the excess mortality attributable to them. We apportioned the sources of uncertainty in excess mortality estimates using a variance-decomposition method.

Results: Estimates suggest that excess mortality attributable to heat waves in the eastern United States would result in 200–7,807 deaths/year (mean 2,379 deaths/year) in 2057–2059. Average excess mortality projections under RCP4.5 and RCP8.5 scenarios were 1,403 and 3,556 deaths/year, respectively. Excess mortality would be relatively high in the southern states and eastern coastal areas (excluding Maine). The major sources of uncertainty were the relative risk estimates for mortality on heat wave versus non–heat wave days, the RCP scenarios, and the heat wave definitions.

Conclusions: Mortality risks from future heat waves may be an order of magnitude higher than the mortality risks reported in 2002–2004, with thousands of heat wave–related deaths per year in the study area projected under the RCP8.5 scenario. Substantial spatial variability in county-level heat mortality estimates suggests that effective mitigation and adaptation measures should be developed based on spatially resolved data.

Citation: Wu J, Zhou Y, Gao Y, Fu JS, Johnson BA, Huang C, Kim YM, Liu Y. 2014. Estimation and uncertainty analysis of impacts of future heat waves on mortality in the eastern United States. Environ Health Perspect 122:10–16; http://dx.doi.org/10.1289/ehp.1306670

## Introduction

Heat is a well-known natural hazard. During the summer, high temperatures may result in heat exhaustion, heat syncope, heat stroke, and heat cramps in susceptible individuals, leading to excess mortality at the population level (Gasparrini and Armstrong 2011; Kovats and Hajat 2008). Heat waves, commonly defined as a few consecutive days with high temperature above a certain threshold, are the leading cause of weather-related mortality in the United States (Davis et al. 2003). For example, the severe heat wave in Chicago, Illinois, in July 1995 resulted in approximately 700 deaths (Semenza et al. 1996). In California, the estimated excess mortality attributable to heat waves in July 2006 ranged from 160 to 333 deaths (Ostro et al. 2009).

According to the Intergovernmental Panel on Climate Change (IPCC), air temperature is projected to rise by 1.8–6.4°C by 2100 (IPCC 2007). The frequency, intensity, and duration of heat waves will likely increase in the future, thus aggravating heat-related mortality unless population adaptation occurs. Consequently, preparedness for adverse outcomes of future heat waves is necessary. However, accurately estimating the health impact of heat waves is challenging because of the uncertainties related to future temperature projections, heat wave metrics, population growth patterns, population susceptibility to heat waves, and spatial heterogeneity of heat waves. A recent study that considered several different climate change scenarios and seven global climate models estimated that future heat waves in Chicago would cause 166–2,217 deaths/year during 2081–2100 (Peng et al. 2011). However, Peng et al. (2011) projected future heat wave mortality in only one city and did not consider spatially resolved temperature data. Because the health impacts of heat waves can have significant spatial variability, mitigation strategies are unlikely to be universally effective. To take local needs into account, spatially resolved estimates of health outcomes due to heat waves are very important.

In the present study, we estimated county-level excess mortality attributable to heat waves across the eastern United States during 2057–2059 using spatially and temporally resolved regional climate model simulation results and also examined factors that contributed to uncertainty. First, we used state-of-the-art high-resolution climate projections to estimate future heat waves at the county level. Next, we estimated the future excess mortality related to heat waves considering several population growth patterns. Finally, we attributed the uncertainties in the excess mortality estimates to various factors using a variance-decomposition method.

## Methods

*Study area*. We focused on the eastern United States (east of 95° longitude), an area that covers approximately 1,700 counties, has a large population (> 180 million), and has diverse weather conditions and geography.

*Climate model simulation data*. Developed at the National Center for Atmospheric Research (NCAR), the Community Earth System Model (CESM1.0) is a coupled climate model that simultaneously simulates the Earth’s atmosphere, ocean, land surface, and sea ice (Gent et al. 2011). For the present study, we used CESM-projected coarse-resolution temperature data for 2057–2059 under two Representative Concentration Pathway (RCP) emissions scenarios. RCPs, the most recent approach to emissions trajectories used by the IPCC (Inman 2011; Moss et al. 2010), include four emissions scenarios (RCP8.5, RCP6.0, RCP4.5, and RCP2.6), representing the radiative forcing levels of greenhouse gases (GHGs) and other forcing agents in 2100. To capture the range of the possible future climate conditions for the present analysis, we used the RCP4.5 scenario, a low-medium scenario of climate change that assumes moderate emissions and the use of a range of technologies and strategies for reducing GHG emissions (Thomson et al. 2011), and the more extreme RCP8.5 scenario, which assumes fossil fuel–intensive energy consumption, with increasing GHG emissions (van Vuuren et al. 2011). The 2050s are generally regarded as the mid-range of climate projection and can potentially be used to capture enough climate change signal while avoiding excessive uncertainties associated with projecting too far into the future (Nolte et al. 2008). We selected the last 3 years of the 2050s (2057–2059) in order to capture signals and heat wave impacts for far enough into the decade.

We used the Weather Research and Forecasting (WRF) model to dynamically downscale the CESM projections. Daily temperature data in 2001–2004, including daily average temperature (T_avg_), daily maximum temperature (T_max_), daily minimum temperature (T_min_), and dew point, were generated by WRF on a 4-km × 4-km grid in the study area. A detailed description of the CESM/WRF modeling was reported previously by Gao et al. (2012). We aggregated 251,262 4-km × 4-km grid cells to 1,703 counties to match population projection data and reduce computational demands. We determined the population-weighted centroid for each county and then averaged the data in the nine grid cells closest to the centroid. To reduce the bias of the WRF temperature simulation results, we used the weather station observations collected in the Meteorological Assimilation Data Ingest System (MADIS) (see Supplemental Material, Figure S1) as the reference to calibrate the WRF temperature data. Specifically, we first calculated the difference between the MADIS and WRF data at each station each day, and then excluded the data points beyond the 99th percentile of the daily temperature data in the MADIS stations. For counties with more than one MADIS station, we averaged the MADIS and WRF data and then obtained the ratios of WRF to MADIS data in each county. We interpolated the calibration ratios from 625 counties with MADIS stations to the 1,703-county study area using either a fixed search radius or flexible search radii. For estimates based on a fixed search radius, we derived county-level calibration ratios based on the average of all calibration ratios in a 150-km radius centered on the population-weighted centroid of each county. The 150-km search radius was selected to ensure that there would be at least one calibration ratio in every search radius. For the second method, we identified the five calibration ratios closest to the county centroid and used the average of these ratios as the calibration ratio for each county.

*Heat wave definitions*. Because there is no universally accepted definition of a heat wave, we used slightly modified versions of four frequently used heat wave definitions (HWDs) (Table 1) to project the frequency and duration (in days) of heat waves from 1 May through 31 September of each year at baseline (2001–2004) and in the future (2057–2059). Specific criteria vary among the definitions, but in brief, the first heat wave definition (HWD_HI) is based on daily low and high heat index values (Robinson 2001), the second (HWD_Tavg) is based on the number of days with a T_avg_ above the 95th percentile (Anderson and Bell 2011), the third (HWD_Tmax) is based on T_max_ values (Meehl and Tebaldi 2004; Peng et al. 2011), and the fourth (HWD_Tmin) is based on the number of days with a T_min_ above the 95th percentile.

**Table 1:** Heat wave definitions used as the basis for the present analysis.

Type	Name	Heat wave criteria	References
HWD_HI	Heat index–based definition	Days of which the low and the high daily heat index (Hi) are no less than the NWS thresholds of 26.7°C (80°F) and 40.5°C (105°F), respectively.	Robinson 2001
HWD_Tavg	Daily average temperature–based definition	At least 2 consecutive days with daily mean temperature (T_avg_) > 95th percentile of T_avg_ during 2001–2004 in summer.^*a*^	Anderson and Bell 2011
HWD_Tmax	Daily maximum temperature–based definition	A heat wave meets three criteria: *a*) daily maximum temperature (T_max_) > 97.5th percentile of T_max_ of summer days during 2001–2004 for at least 3 days, *b*) the average of T_max_ greater than this threshold for the entire period, and *c*) T_max_ > 81.5th percentile of T_max_ of summer days during 2001–2004 for every day during the entire period.^*a*^	Meehl and Tebaldi 2004; Peng et al. 2011
HWD_Tmin	Daily minimum temperature–based definition	At least 2 consecutive days with daily minimum temperature (T_min_) > 95th percentile of T_min_ during 2001–2004 in summer.^*a*^	Zhang et al. 2012
NWS, National Weather Service. ^***a***^The definitions of heat waves were modified for the present analysis because we used temperature data collected ­during 2001–2004 (from 1 May through 31 September of each year) as the baseline data.			

According to HWD_Tavg, some days with relatively mild temperatures might be counted as heat wave days in counties with a low T_avg_ during May through September. Therefore, we modified the original definition used by Anderson and Bell (2011) by setting 26.7°C (80°F) as the minimum threshold of T_avg_ used to define a heat wave. For the same reason, we set 21.3°C (70.8°F) as the T_min_ threshold, and 32.7°C (90.9°F) as the T_max_ threshold, when defining heat waves based on HWD_Tmin or HWD_Tmax, respectively. These thresholds were selected because the average T_min_ and T_max_ differ from the average T_avg_ from 1 May–31 September by 5.4°C and 6°C in our data set, respectively (see Supplemental Material, Figure S2).

*Future population projections*. We based our estimates of county-level populations for 2057–2059 on state-level projections conducted by the U.S. Census Bureau using the cohort-component method (U.S. Census Bureau 2009), in which the components of population change (births, deaths, and net international migration) were projected from the 2000 base population to the year 2050 for each birth cohort (Preston et al. 2001). To conduct this projection, the Census Bureau used multiple data sources to generate information about fertility, mortality, and migration. In addition, the Census 2009 projection incorporated four net international migration assumptions: *a*) high migration, *b*) constant/medium migration, *c*) low migration, and *d*) zero migration. For the present analysis, we projected future populations at the county level using the constant ratio method (Shryock et al. 1973). Specifically, for each county, we multiplied the census-projected state population in 2050 by the ratio of the county population to the state population at baseline in 2000. The approach assumes that each county’s share of the state population remains constant over time.

*Estimation of heat wave mortality*. The expected number of excess deaths attributable to heat waves (*ED*_hw_) in each county was calculated using Equation 1 (Peng et al. 2011):

*ED*_hw_ = *N* × (*RR* – 1) × *L*, [1]

where *N* is the expected number of deaths on non–heat wave days in each county, which is equal to the expected daily mortality rate on non–heat wave days multiplied by county population, and *RR* is the relative risk of death on heat wave days compared with non–heat wave days. *RR* – 1 is the attributable risk (AR) to heat waves (i.e., the increase in the risk of nonaccidental mortality on heat wave days compared with non–heat wave days), and *L* is the length of the heat wave in days.

Because nearly 99% of counties had no heat waves in 2001 by our definitions, we used the county-level nonaccidental daily mortality rate in 2001 as the expected daily mortality rate on non–heat wave days during the 2002–2004 baseline period. Similar to Peng et al. (2011), we assumed that the nonaccidental mortality rate on non–heat wave days in 2057–2059 was unchanged from the baseline rate. We used the 95% confidence interval (CI) estimated by Anderson and Bell (2011) for the percentage increase in nonaccidental mortality on heat wave days compared with non–heat waves days during 1987–2005 to define the ranges of possible AR values for counties in the Northeast (1.79–11.98%), the Midwest (3.36–7.93%), and the South (–0.11 to 3.84%). These estimates reflect estimated excess risks of mortality on heat wave days defined using HWD_Tavg (Table 1). Based on the geographic location of each state, the Northeast region includes Connecticut, Maine, Massachusetts, New Hampshire, New Jersey, New York, Pennsylvania, Rhode Island, and Vermont. The Midwest region includes Illinois, Indiana, Iowa, Kansas, Michigan, Minnesota, Missouri, Nebraska, Ohio, and Wisconsin. The South region includes Alabama, Arkansas, Delaware, Florida, Georgia, Kentucky, Louisiana, Maryland, Mississippi, Oklahoma, North Carolina, South Carolina, Tennessee, Texas, Virginia, Washington, DC, and West Virginia. We generated 100 random samples of AR values from uniform distributions based on the ranges for each region using Latin hypercube sampling, a modified Monte Carlo simulation method (Helton and Davis 2003) and randomly selected nine of the sampled sets of values (each of which included three AR values, one for each region—the counties in each region have the same AR values) to use in our final calculations.

*Uncertainty and sensitivity analysis*. We considered the factors contributing to the uncertainties in projected heat wave health impacts in each analytical step. These factors include the two RCP scenarios (RCP4.5 and RCP8.5), the two methods used to calculate the temperature calibration ratios for each county (fixed search radius and flexible search radii), the four heat wave definitions (Table 1), the four population projections (assuming high, constant/medium, low, or zero net migration), and the nine sets of sampled AR values. In addition, we estimated excess mortality due to heat waves for 1,703 individual counties in each of 3 future years (2057–2059). After calculating the total excess mortality attributable to heat waves considering all the sources of variation mentioned above, we derived probability distributions, mean values, SDs, and 95% CIs for an overall estimate and for subsets of estimates according to different assumptions (e.g., according to RCP scenarios and heat wave definitions).

We used the variance-decomposition method in our sensitivity analysis of the influence of different factors on heat wave mortality estimates because it not only identifies influential factors but also apportions the sources of uncertainty (Chan et al. 1997; Saltelli et al. 2008). For a generic model, *y = f*(*x*1, *x*2*,…xn*), the total variance of *y* can be decomposed into the partial variance attributable to each factor (*x*1, *x*2,…*xn*) and their interactions. In our analysis, *y* is the excess mortality estimate; *x*1, *x*2,…*xn* are *n* factors related to *y*; and the total variance was decomposed using the following equations:

*V*(*y*) = *V*(*y*|*x*1) + *V*(*y*|*x*1, *x*2) +…*V*(*y*|*x*1, *x*2,…*xn*) [2]

and

*Si* = *V*(*y*|*xi*)/*V*(*y*), [3]

where *V*(*y*) is the total variance of *y*, *V*(*y|x*1) is the variance of *y* attributable to *x*1, and *V*(*y*|*x*1,*x*2,…*xn*) is the variance of *y* attributable to the interactions of *x*1, *x*2,*…xn*. *Si*, the first-order sensitivity index for factor *i*, reflects the main effect of each factor on the estimate of the heat wave mortality. A large value of *S* for a given factor indicates that the estimate of excess mortality is more sensitive to this factor than factors with a smaller *S*. We identified factors that have larger influences on the total excess mortality of the whole study area (which are the main sources of uncertainty in the estimation of heat wave mortality).

## Results

*Characteristics of heat waves*. After calibrating the WRF temperature simulations to observed temperatures in MADIS using the calibration method based on a fixed search radius (the distribution of calibration ratios is shown in the Supplemental Material, Figure S3), the estimated T_avg_ (± SD) over the entire study area during May–September over both RCP scenarios was 22.74°C (± 4.67) during 2002–2004 and 24.75°C (± 4.46) during 2057–2059 (Table 2). Average estimated numbers of heat waves per county per year were 0.38 ± 0.91 during 2002–2004 and 1.88 ± 3.11 during 2057–2059. Temperatures projected for 2057–2059 based on the RCP8.5 scenario were nearly 1°C higher than projections based on the RCP4.5 scenario, resulting in almost twice as many projected heat waves. The projected average duration of heat waves in 2057–2059 based on both RCP scenarios (4.53 ± 3.09 days) is nearly 1 day longer than the estimated average duration in 2002–2004 (Table 2).

**Table 2:** Estimated current (2002–2004) and future (2057–2059) heat waves and related excess mortality (mean ± SD).

Period	Daily average temperature (°C)^*a*^	Heat wave frequency (episodes/year)^*b*^	Heat wave duration (days)^*b*^	Total excess deaths/year (95% CI)^*c*^
2002–2004	22.74 ± 4.67	0.38 ± 0.91	3.44 ± 1.78	187 ± 173 (2, 614)
2057–2059^*d*^	24.75 ± 4.46	1.88 ± 3.11	4.53 ± 3.09	2,379 ± 2,008 (200, 7,808)
2057–2059 (RCP4.5)	24.11 ± 4.30	1.31 ± 2.57	4.06 ± 3.31	1,403 ± 1,015 (137, 3,788)
2057–2059 (RCP8.5)	25.39 ± 4.51	2.44 ± 3.49	4.85 ± 3.49	3,556 ± 2,265 (300, 8,577)
^***a***^During May through September in 1,703 eastern U.S. counties. ^***b***^Integrated averages obtained using four heat wave definitions. ^***c***^Integrated averages obtained using four heat wave metrics and four population projections. ^***d***^Values shown are integrated over the two RCP scenarios, and the total excess deaths account for all possible excess relative risk values.				

When estimated using only one heat wave definition at a time, HWD_HI resulted in the smallest estimated numbers of future heat waves, and HWD_Tmin resulted in the largest numbers (Table 3). HWD_Tmax produced the longest estimate of average heat wave duration (4.88 ± 2.98 days), whereas projections based on the other three heat wave definitions were between 3 and 4 days. Average heat wave durations estimated under the RCP8.5 scenario were almost 1 day longer than under the RCP4.5 scenario, regardless of the heat wave definition used (Table 3).

**Table 3:** Projected average heat wave days and episodes per year during 2057–2059 in each county according to emission scenario (RCP4.5 or RCP8.5) and heat wave definition (mean ± SD).

Heat wave metrics	Heat wave frequency (episodes/year/county)		Heat wave duration (days)	
	RCP4.5	RCP8.5	RCP4.5	RCP8.5
HWD_HI	0.49 ± 1.80	0.79 ± 2.46	3.29 ± 1.83	4.00 ± 3.63
HWD_Tmax	0.50 ± 0.88	1.50 ± 1.75	4.88 ± 2.98	5.80 ± 4.31
HWD_Tavg	1.84 ± 2.54	3.40 ± 3.37	3.96 ± 2.36	4.69 ± 3.57
HWD_Tmin	2.43 ± 3.61	4.06 ± 3.58	3.75 ± 1.47	4.33 ± 1.97

**Figure 1:**
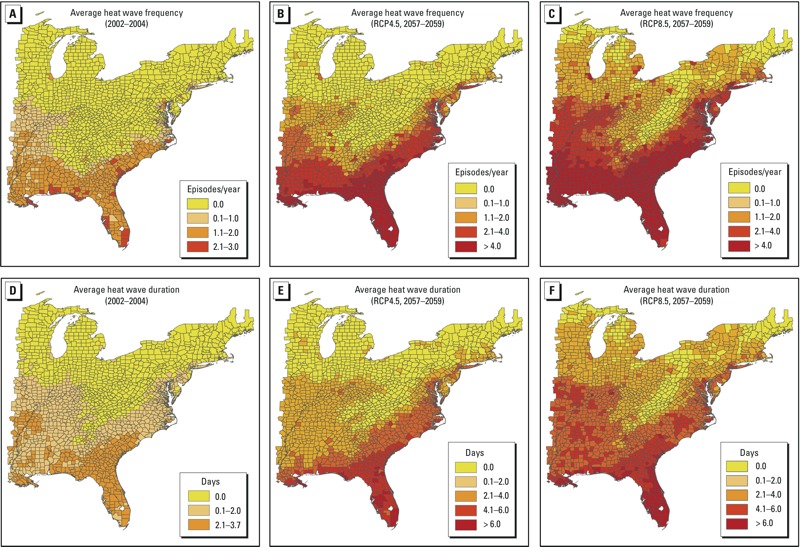
Baseline (2002–2004; *A*,*D*) and future (2057–2059; *B*,*C*,*E*,*F*) estimates of heat wave frequency and duration in study area counties. Heat wave frequency and duration estimates are integrated averages based on four heat wave metrics.

Projected average values for the duration and frequency of heat waves in 2057–2059 (integrated over the four heat wave definitions) suggest that there will be considerable spatial variability at the county level (Figure 1). In particular, most counties in the northern half of the study region would not experience any heat waves under the RCP4.5 scenario (Figure 1B), whereas most counties along the southern coast would average ≥ 2/year, and Florida counties would average ≥ 4 heat waves/year. Under the RCP8.5 scenario, the number of non–heat wave counties in the Northern region would decrease, and the average frequency of heat waves in most of the counties in the southern states including Alabama, Florida, Georgia, Louisiana, and South Carolina would increase to ≥ 4/year (Figure 1C). Counties in southern Florida have the longest average heat wave durations projected for the study area under both RCP scenarios (Figure 1E,F).

*Future heat wave mortality*. The estimated average number of heat wave deaths in the eastern United States during 2002–2004 was 187 ± 173 deaths/year, in contrast with 2,379 ± 2,008 deaths/year projected for 2057–2059 (integrated over four population projections, four heat wave definitions, and two RCP scenarios) (Table 2). Projected numbers of deaths differ substantially between the two RCP scenarios, with a mean of 1,403 ± 1,015 deaths/year projected under RCP4.5, compared with 3,556 ± 2,265 deaths/year under RCP8.5. Projections also differ according to the heat wave definition used (Table 4), with almost 2.5 times more deaths projected based on HWD_Tavg and HWD_Tmin than estimated using the other two definitions, for both RCP scenarios. As expected, estimated numbers of heat wave deaths increase as projected populations increase in size due to migration (Table 4).

**Table 4:** Projected excess mortality per year attributable to heat waves during 2057–2059 under different heat wave definitions, population projections, and emission scenarios (mean ± SD).

Factor	Category	RCP4.5	RCP8.5
Heat wave definitions	HWD_HI	898 ± 509	1,668 ± 936
	HWD_Tmax	707 ± 378	2,496 ± 1,515
	HWD_Tavg	1,953 ± 1,033	4,499 ± 2,274
	HWD_Tmin	2,053 ± 1,091	4,759 ± 2,315
Population projection	Extreme high	1,568 ± 1,118	3,746 ± 2,484
	High	1,472 ± 1,048	3,517 ± 2,334
	Low	1,412 ± 1,006	3,377 ± 2,242
	Extreme low	1,159 ± 826	2,783 ± 1,854

**Figure 2:**
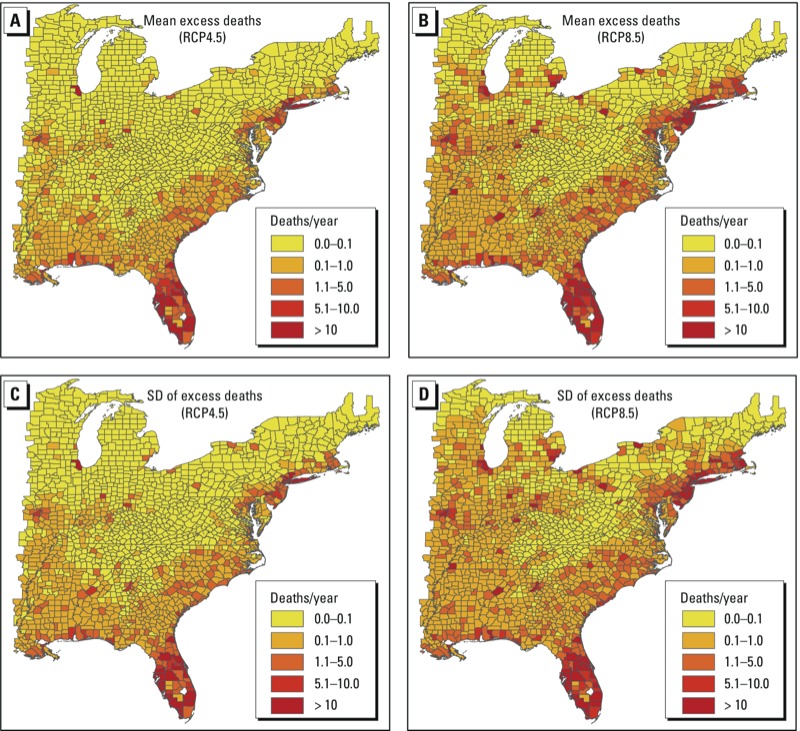
Projected county-level excess deaths [means (*A*,*B*) and SDs (*C*,*D*)] due to heat waves in 2057–2059 under RCP4.5 (*A*,*C*) and RCP8.5 (*B*,*D*) scenarios. The excess deaths for each scenario are average estimates obtained using four heat wave metrics and four population projections.

Our excess mortality estimates have substantial spatial variability (Figure 2). Under the RCP4.5 scenario (Figure 2A,C), the highest county-level excess mortality estimates (> 10 deaths/year) were projected for counties in Florida, New York, and Illinois. Under the RCP8.5 scenario (Figure 2B,D), > 10 deaths/year are projected for a larger number of counties in Florida in addition to some counties along the East Coast (e.g., in Massachusetts, New York, and New Jersey) and a few counties in inland states including Illinois and Michigan. The high numbers of projected deaths in metropolitan areas of Illinois, Massachusetts, Michigan, New Jersey, and New York might be associated with the high population density in these areas (see Supplemental Material, Figure S4).

**Figure 3:**
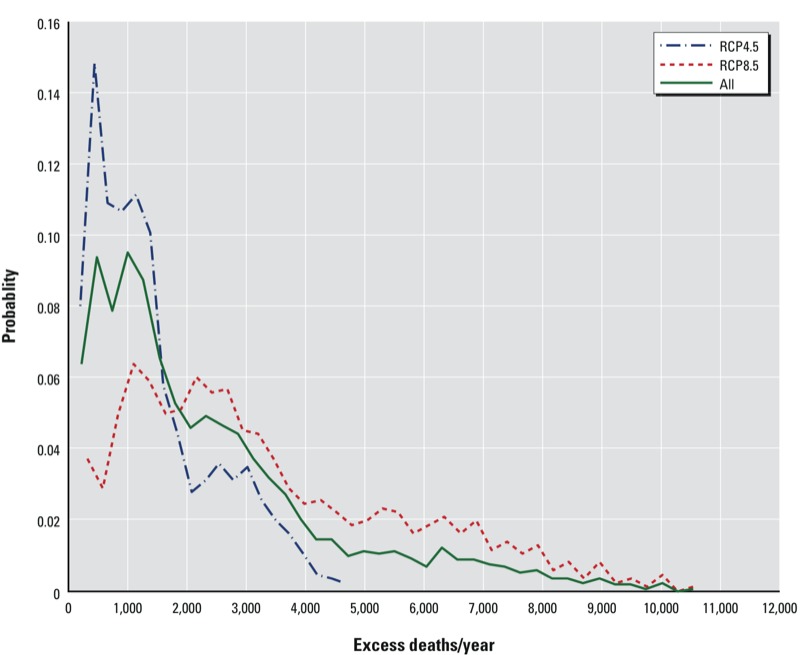
Probability distribution of annual excess mortality attributable to heat waves in the study domain under RCP4.5, RCP8.5, and the two scenarios combined. Data shown were derived from pooling the total excess deaths under all possible situations considered in this study, including two temperature calibration methods, four heat wave definitions, four population projections, 3 years, and nine sets of AR values.

*Uncertainty and sensitivity analysis*. Figure 3 shows the probability distributions of annual total excess mortality estimates in the eastern United States for 2057–2059 under RCP4.5, RCP8.5, and the two scenarios combined. All the factors that contribute to the uncertainty in estimated excess deaths were accounted for, including two temperature calibration methods, four heat wave definitions, four population projections, three model years, and nine sets of AR values. Under the RCP4.5 scenario, the estimated number of deaths is 1,403 deaths/year (95% CI: 137, 3,788). The distribution curve under this scenario peaks at around 500 deaths/year, then drops rapidly. The cumulative probabilities of < 1,000 excess deaths/year and < 2,000 excess deaths/year are 43% and 76%, respectively; we saw no probability of > 5,000 excess deaths/year. In contrast, the probability distribution of estimated excess deaths under RCP8.5 is relatively flat, with a cumulative probability of 42% for having 1,000–3,000 excess deaths/year, and 15% for having 5,000–7,000 excess deaths/year, and a very small probability for having > 10,000 excess deaths/year. When estimates are integrated over both RCP scenarios, the probabilities of different excess mortality estimates fall between those of the scenario-specific estimates.

**Figure 4:**
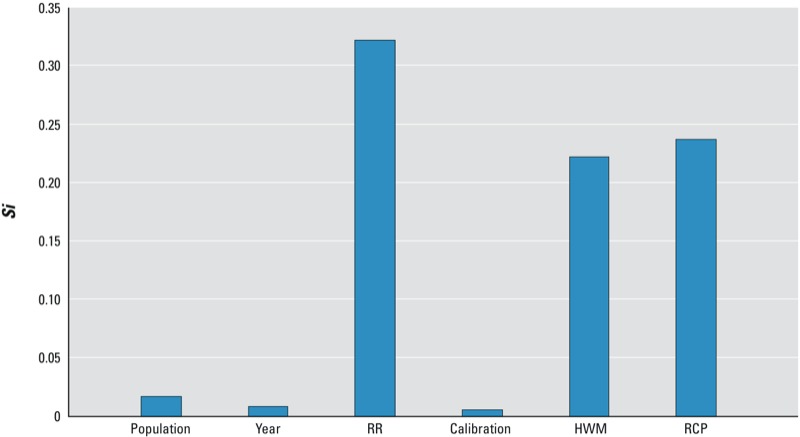
Sensitivity analysis of annual heat wave mortality affected by various factors. The factor with a higher *Si* value has higher influence on estimated the excess mortality. Abbreviations: RR, the relative risk of heat wave days to non–heat wave days; calibration, calibration ratio interpolation methods; HWM, heat wave metrics; RCP, RCP scenarios.

Using the variance-decomposition method, we attributed 23.7% of the uncertainty to the two RCP scenarios, 22.2% to the four heat wave definitions, and 32.2% to the different values for the relative risk of mortality on heat wave days compared with non–heat wave days [including nine randomly sampled sets of three area-specific ARs based on previously published estimates (Anderson and Bell 2011)]. In contrast, the two methods used for the WRF data calibration, the four migration scenarios used in the population projections, and the interannual variability of temperature projections during 2057–2059 were not major sources of uncertainty, as indicated by their low *Si* values (Figure 4).

## Discussion

We estimated that heat waves will be 3.5–6.4 times more frequent in 2057–2059 than in 2002–2004 and that excess mortality attributable to heat waves would be 7.5–19.0 times higher. The major sources of uncertainty in our heat wave mortality projections were the RCP scenarios, the ARs of mortality on heat wave days compared with non–heat wave days, and the criteria used to define heat waves.

In contrast with previous studies that only focused on one or a few cities (Peng et al. 2011; Sheridan et al. 2012), we estimated the impacts of future heat waves over a wide geographic area. Our estimates suggest that there will be high spatial variability in future heat wave mortality and that estimates of climate change health impacts based on a single geographic area will have limited value for estimating health impacts in other areas. In addition, we performed a comprehensive examination of six factors that may contribute to uncertainty in heat wave mortality projections, whereas previous studies explored a relatively limited set of factors [e.g., GHG emission scenarios (Peng et al. 2011)]. Furthermore, we used recently developed RCPs (Inman 2011; Moss et al. 2010) for temperature projections at a high spatial resolution, whereas most previous studies (Kolstad and Johansson 2011; Peng et al. 2011) were based on older emission scenarios described in the *IPCC Special Report on Emissions Scenarios* (IPCC 2000).

We estimated that there would be an average of 1.88 (95% CI: 0, 11) heat wave episodes per year per county in 2057–2059. The projected geographic distribution of heat waves has considerable spatial variability, with 35.7% of counties expected not to experience any heat waves, whereas 10.4% of counties would experience > 4 heat waves/year under the RCP4.5 scenario, and corresponding estimates of 11.6% and 26.5% under the RCP8.5 scenario, respectively. Heat waves would be expected to occur most often in the southern coastal states, including Florida, Georgia, and Louisiana, with Florida counties having the highest numbers of deaths attributable to heat waves. In addition, our projections suggest that despite relatively low frequencies of heat waves, heat wave mortality will be high in densely populated counties located in several northeastern coastal states (e.g., Massachusetts, New Jersey, and New York).

Increases in future temperatures will depend largely on GHG emissions. Therefore, GHG emission scenarios may have a substantial influence on estimates of the frequency and duration of future heat waves. We estimated future heat waves under two scenarios representing moderate and high GHG emissions. Under the high-emission RCP8.5 scenario, excess mortality was estimated to be almost 2.5 times higher than that estimated under the moderate RCP4.5 scenario, suggesting that curtailing GHG emission will have a great impact on the reduction of heat wave mortality in the future.

Our findings also indicate that heat wave definitions have a strong influence on heat wave mortality projections, with the fewest days per year being classified as heat wave days when the HDW_HI definition was used, and the largest numbers of heat wave days when heat waves were defined using HDW_Tavg or HDW_Tmin. Our results are consistent with recent studies that reported substantial inconsistencies among heat wave mortality estimates when using different heat wave definitions (Hajat et al. 2010; Zhang et al. 2012).

Estimates of the relative risk of mortality on heat wave days compared with non–heat wave days have varied among previous studies and may at least partly reflect regional differences in susceptibility to adverse effects of heat at the population level as well as individual-level differences in susceptibility according to race, age, occupation, or other factors (O’Neill et al. 2005). Currently, there is little information about regional variation in the relative risk of heat wave mortality; therefore, we used a Monte Carlo simulation to sample AR from values previously reported by Anderson and Bell (2011) for different regions of the United States. Although this allowed us to estimate excess mortality based on an appropriately wide range of excess relative risk estimates, it also contributed substantial uncertainty to our estimates. In addition, we considered AR estimates reported by Bobb et al. (2011) for heat waves defined based on a T_max_, in contrast with estimates reported by Anderson and Bell (2011), who defined heat waves based on a T_avg_. In general, AR values estimated by the two groups were similar except for the Midwest region, where the estimates reported by Anderson and Bell (2011) were slightly higher. We sampled the values of excess relative risks for the three regions of our study area from values spanning the 95% CIs of corresponding ARs reported by Anderson and Bell (2011) because their estimates had wider ranges, thus representing more conservative uncertainty estimates in the calculated excess deaths due to heat waves.

Our study has several limitations. Our estimates of heat wave impacts on mortality are primarily based on temperatures in urban areas, where the majority of eastern U.S. residents live. However, heat stress responses may differ between urban and rural populations (Fischer et al. 2012). Urban temperatures are generally higher than temperatures in surrounding rural areas, especially at night, because of the urban heat island effect. Therefore, the relative risk of heat-related mortality may be much higher in urban areas than in surrounding rural areas (Clarke 1972). If lower impacts of heat waves on rural populations had been accounted for in our analysis, our estimates of annual mortality due to heat waves would have been slightly smaller.

Our estimates did not include heat-related deaths on hot days that were not identified as heat wave days according to our heat wave definitions. In addition, our estimates did not account for higher temperatures during future heat waves, which will be hotter than those during heat waves in 2002–2004, even under the RCP4.5 scenario (Table 2). To better account for the temperature effect, a national-scale epidemiological study is needed to provide region-specific, parametric exposure-response functions between temperature and mortality.

We also did not consider effects of human adaptation or heat mitigation measures on future heat wave mortality. Similar to Peng et al. (2011), we assumed that excess mortality from future heat waves will be the same as heat wave mortality at baseline. However, if mitigation measures to prevent heat-related mortality are adopted as heat waves become more frequent, excess mortality would decrease, and our projections would overestimate future heat-related mortality in some areas. Heat warning systems and air conditioning have been reported to reduce heat wave impacts (Anderson and Bell 2009; Fouillet et al. 2008; O’Neill et al. 2009). Other measures such as planting trees can also reduce temperatures, thus reducing heat-related mortality (Akbari 2002). Finally, our population projections did not consider demographic changes (e.g., changes according to race/ethnicity, sex, and age) that might have considerable impacts on heat wave mortality (Hajat and Kosatky 2010; O’Neill et al. 2005).

## Conclusion

Our results suggest that numbers of heat wave–related deaths are likely to be an order of magnitude higher in 2057–2059 than in 2002–2004. Under the fossil-fuel–intensive RCP8.5 scenario, the probability of thousands of heat wave–related deaths per year in the eastern United States is much higher than under the RCP4.5 scenario. Effective mitigation and adaptation measures will be crucial to reduce the potential for catastrophic outcomes, particularly in the most vulnerable geographic regions. In addition, we found that heat wave definitions, GHG emission scenarios, and estimates of the relative risk of mortality on heat wave days compared with non–heat wave days account for a large proportion of the total variation in projected mortality estimates and are major sources of uncertainty.

## Supplemental Material

(7.5 MB) PDF
