# Supplementary material for: Estimation and Uncertainty Analysis of Impacts of Future Heat Waves on Mortality in the Eastern United States
Source: Environ Health Perspect. 2013 Nov 6;122(1):10–6. doi: 10.1289/ehp.1306670 (PMC3888568; doi:10.1289/ehp.1306670)
Supplement: (7.5 MB) PDF [file ehp.1306670.s001.508.pdf]

Environmental Health Perspectives doi:10.1289/ehp.1306670

Supplemental Material

Estimation and Uncertainty Analysis of Impacts of Future Heat Waves on Mortality in the Eastern United States

Jianyong Wu, Ying Zhou, Yang Gao, Joshua S. Fu, Brent A. Johnson, Cheng Huang, Young-Min Kim, and Yang Liu

Table of contents

Figure S1. The spatial distribution of the MADIS (Meteorological Assimilation Data Ingest System) station and calibration ratios.......…………………………………………..….2

Figure S2. The average daily temperature during the warmest months of the year (May 1-September 31)…………………………………………………………………3

Figure S3. The distribution of calibration ratios between WRF temperature and MADIS temperature based on the fixed-search-radius calibration method……..........4

Figure S4. The spatial distribution of population in 2000 and 2050………………………..5

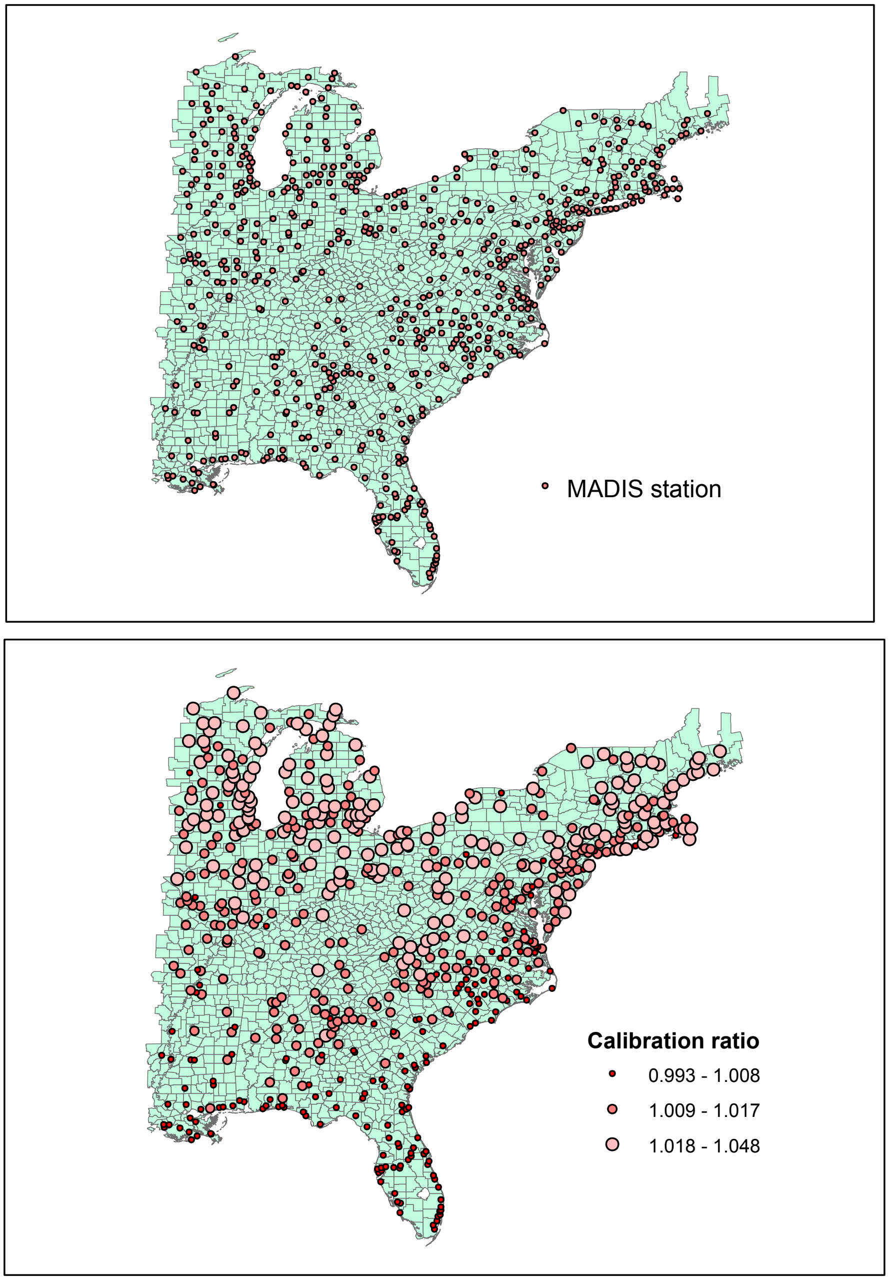

Figure S1. The spatial distribution of the MADIS (Meteorological Assimilation Data Ingest System) station and calibration ratios. The calibration ratios were the ratios of the average temperature obtained from the Weather Research and Forecasting (WRF) model to the average temperature obtained from MADIS station in a county in a same time period.

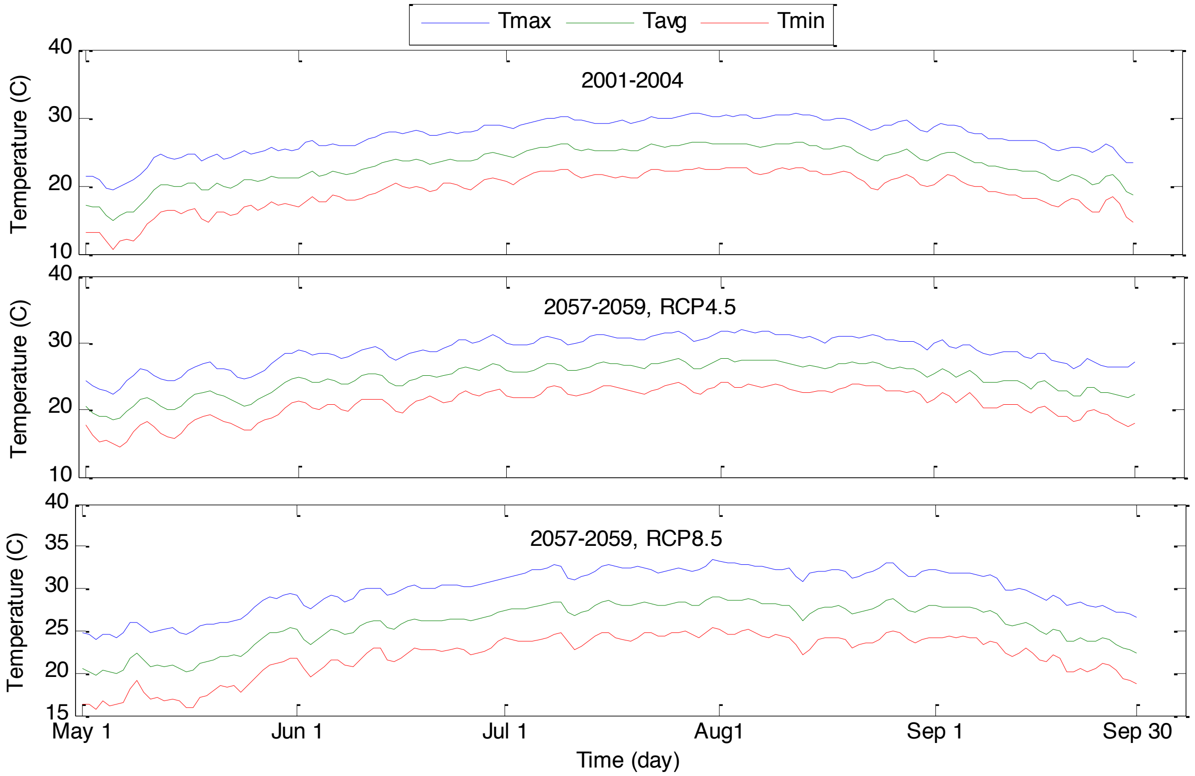

Figure S2. The average daily temperature during the warmest months of the year (May 1-September 31). The temperature data are averaged over all the counties in the study area and 3 years. Here, Tmax: daily maximum temperature; Tavg: daily average temperature; Tmin: daily minimum temperature. Based on the temperature data from May 1 - September 31 during 2001-2004, the mean difference between the daily minimum temperature and the daily average temperature was 5.4°C, and the mean difference between the daily average temperature and the daily maximum temperature was 6°C.

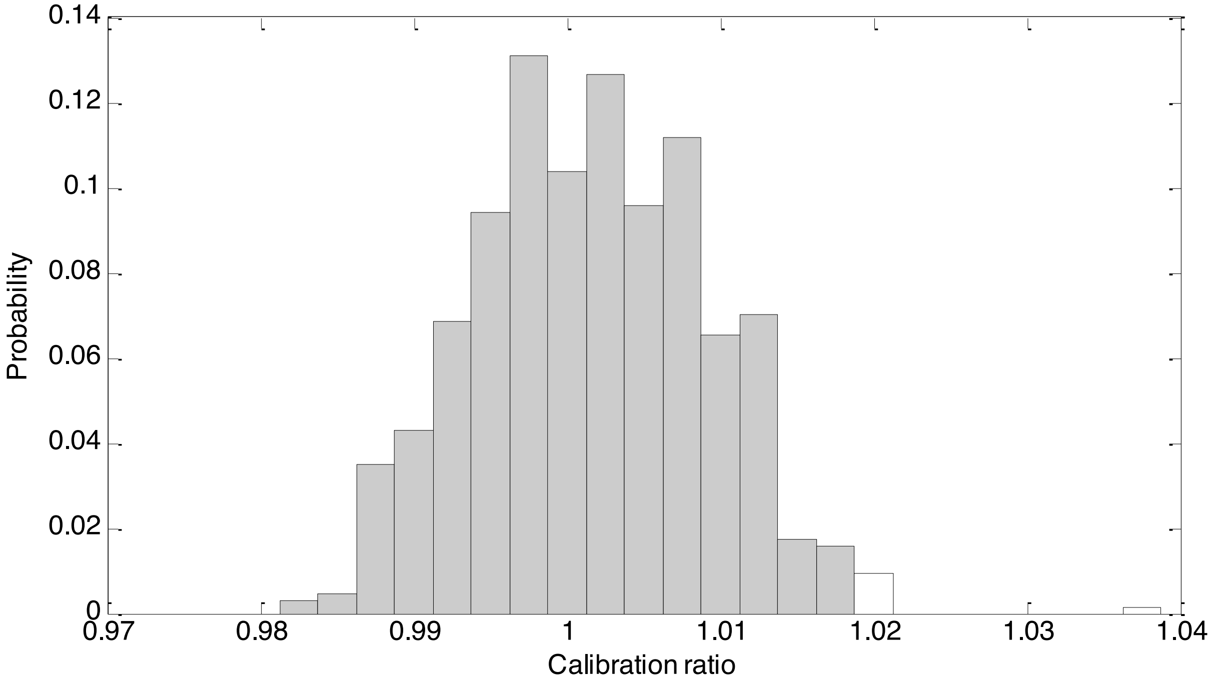

Figure S3. The distribution of calibration ratios between WRF temperature and MADIS temperature based on the fixed-search-radius calibration method. The calibration ratios based on the flexible-search-radii calibration method show the same distribution. The temperature data measured from 625 MADIS stations were used as the reference to calibrate the data generated by the WRF model. The results indicated the projected data matched the monitoring data very well. The ratios between two datasets are close to 1.000 with a 95% confidence interval of 0.999-1.028.

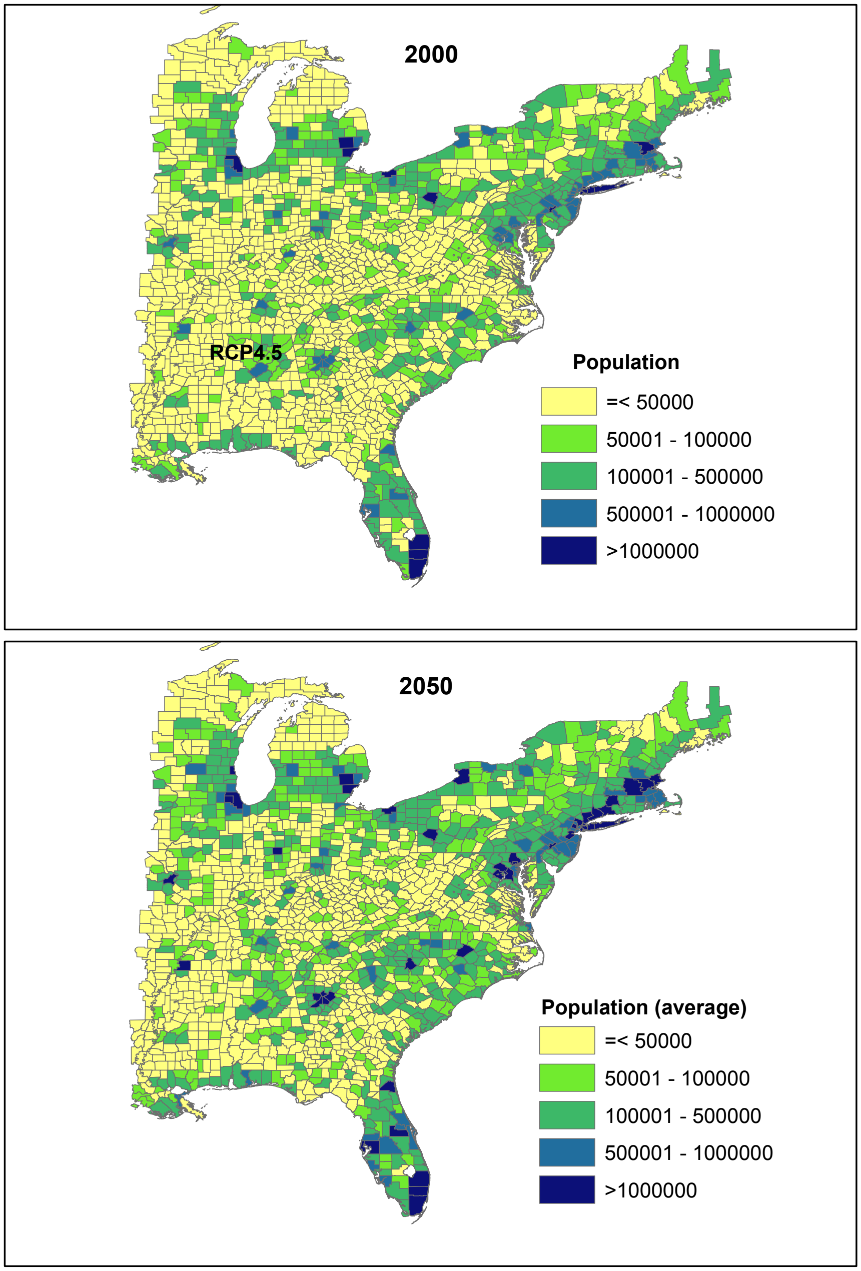

Figure S4. The spatial distribution of population in 2000 and 2050. The population in 2050 demonstrated in this map was the average of four projections, which incorporate four alternative net international migration assumptions: 1) high migration, 2) constant/medium migration, 3) low migration, and 4) zero migration. The population in each county in 2050 was obtained by

multiplying the projected state population in 2050 by the ratio of the county population to the state population at baseline in 2000.
